# Few Runners Return to Running after Total Joint Arthroplasty, While Others Initiate Running

**DOI:** 10.5435/JAAOSGlobal-D-23-00019

**Published:** 2023-04-19

**Authors:** Brielle Antonelli, Rebecca Teng, Rebecca G. Breslow, Matthew Jamison, Matthew Hepinstall, Ran Schwarzkopf, Wayne E. Moschetti, Antonia F. Chen

**Affiliations:** From the Department of Orthopedic Surgery, Brigham and Women's Hospital, Harvard Medical School, Boston, MA (Ms. Antonelli, Dr. Breslow, Dr. Jamison, and Dr. Chen); Force Therapeutics, Inc. New York, NY (Ms. Teng and Ms. Ulcoq); New York University Langone Health, New York, NY (Dr. Hepinstall, Dr. Schwarzkopf, and Dr. Davidovitch); Norwell Health, New Hyde Park, NY (Dr. Scuderi); Dartmouth-Hitchcock Medical Center, Lebanon, NH (Dr. Moschetti, Dr. Jevsevar, and Dr. Fillingham); Rothman Orthopaedic Institute, Philadelphia, PA (Dr. Grossman and Dr. Ponzio).

## Abstract

This study examines self-reported running practices in total joint arthroplasty (TJA) patients.

**Methods:**

This prospective, cross-sectional study of a multi-institutional database identified 4,462 primary total hip arthroplasty (THA) and total or partial knee arthroplasty (TKA/UKA) patients from June 2015 to June 2020. TJA patients completed an online survey capturing pre-TJA running experience and expectations, surgeon recommendations about return to running, postoperative characteristics, revision surgeries, and the Commitment to Exercise Scale and Brief Resilience Scale. Patient-reported follow-up reached 5 years. Logistic regression, the chi square test, and analysis of variance tests were used.

**Results:**

Five hundred forty-nine patients (12.2%) self-reported running preoperatively, and 65 patients (11.8%) returned to running after surgery. 67.2% were satisfied with their return to running. 40 patients (1.0%) who were not preoperative runners started running after TJA. Preoperative runners who returned to running had higher mean Brief Resilience Scale (3.9 ± 0.7) scores and the highest Commitment to Exercise Scale scores (46.5 ± 17.6, F[2,510] = 3.88, *P* = 0.02). Runners who returned to running had a 6.2% revision rate while those who did not run postoperatively had a 4.8% revision rate (*P* = 0.55). Surgeon recommendations included no return to running (29.5%), maintain low-impact activities (35.2%), return to preoperative levels (5.1%), and no recommendations (30.1%).

**Discussion:**

12% of TJA preoperative runners returned to running, mostly within 1 year, and 67.2% were satisfied with their running ability.

Although the number of total joint arthroplasties (TJAs) conducted in the United States is steadily increasing, patient-reported outcomes for these procedures vary depending on expectations and preoperative activity levels. This may be especially applicable to runners because the force placed on the knees from running is eight times that of the body weight and the hip sustains five times the body weight.^1,2^ However, the ability to return to running after TJA is unknown,^3,4^ especially because the effects of total knee arthroplasty (TKA) on knee kinematics and its ability to withstand the high joint load of running may limit the ability to return to running after surgery. TKA does not reliably restore normal knee kinematics relating to varus/valgus rotation and anterior translation of the femur with early flexion.^5^ This may explain why most TKA patients do not return to high-impact activities, such as jogging, tennis, basketball, soccer, dancing, and alpine skiing.^6^ 26.4% of patients undergoing TKA or total hip arthroplasty (THA) had to decrease their activity level after surgery because of joint pain.^7^ Studies investigating the time to return to low-impact sports in physically active patients found that 62% of THA patients were able to return to sports 1 to 3 years after surgery,^7^ and TKA patients were able to return to sports approximately 4 to 5 years after TKA.^8,9^

Athletes are often advised to avoid high-impact activities after TJA despite the feasibility of returning to sports.^10^ Studies have identified younger age, lower body mass index (BMI),^11^ male sex, and higher University of California Los Angeles activity score^7^ as positive predictive factors for returning to activity after TJA, but other potential factors, such as patient resilience or high motivation to exercise, need investigation.

Investigating the ability of patients undergoing TJA to run after their surgery may help characterize current practices and develop future evidence-based protocols. There were multiple hypotheses for our study. We hypothesized that a small percentage of patients who ran before TJA returned to running after surgery, and no patients started running after TJA. We also hypothesized that injury and revision surgery were higher in patients who returned to running after TJA. In addition, we hypothesized that patients who returned to running after TJA had higher exercise dependency and resilience. Finally, we hypothesized that there is no consensus among surgeons regarding recommendations for returning to running after TJA. Thus, this study aims to 1) identify postoperative running volume and frequency in two cohorts of patients—a) those who ran before TJA and returned to running and b) patients who started running after TJA; 2) characterize self-reported injury incidence and revision surgery rates; 3) examine the personality characteristics of exercise dependency and resilience; and 4) analyze patterns in surgeon recommendations about returning to running after TJA.

## Methods

### Study Patients and Online Platform

A prospective, multisite survey study was conducted that surveyed patients with surgical dates from June 29, 2015, to June 16, 2020, on a standard electronic patient engagement platform (Force Therapeutics). Patients who underwent primary THA, TKA, or unicompartmental knee arthroplasty (UKA) and had an active e-mail to opt in to the platform were eligible for this study. Patients who underwent revision THA or TKA were excluded. This study was approved by the Institutional Review Board.

### Survey Study and Outcomes

Demographics (age, height, weight, sex, and BMI) and surgical information were collected. A survey was designed to target patients who were either runners before undergoing THA/TKA/UKA or who were not runners preoperatively but started running postoperatively (Appendix, http://links.lww.com/JG9/A275). The survey assessed running frequency per week, average weekly mileage, and running intensity. The survey also contained validated assessments for exercise commitment (Commitment to Exercise Scale [CTE]) and resilience (Brief Resilience Scale [BRS]).^12,13^ Higher CTE scores indicated a stronger dependency on exercise (range 0 to 80), and higher BRS scores indicated higher resilience (range 1 to 5), with 1.00 to 2.99 correlating with low resilience, 3.00 to 4.30 with normal resilience, and 4.31 to 5.00 with high resilience.

If patients reported “yes” to returning to running after surgery, they received additional questions to characterize their postoperative running behavior, such as pain, how long it took to return to running, running frequency and intensity, running satisfaction, injury, and revision surgery. Patients completed the survey preoperatively and then postoperatively at 6 weeks, 6 months, 1 year, 2 years, 3 years, 4 years, and 5 years after THA/TKA/UKA. The average patient follow-up period was 12.4 months (range 6 weeks to 5 years).

### Statistical Analysis

Descriptive statistics were completed, and the chi square test and Fisher exact test were used to determine differences in (1) the frequency of patients who returned to running after THA/TKA and (2) the frequency of revision surgery between preoperative runners who returned to running versus preoperative runners who did not return to running. Analyses of variance evaluated differences in mean CTE and BRS scores between the runner cohorts. Univariate logistic regression assessed whether there was a relationship between BRS/CTE scores and postoperative running status for each procedure (SAS Institute). Statistical significance was considered at *P* < 0.05.

## Source of Funding

No funding was used to conduct this study.

## Results

### Demographics

A total of 4483 patients received the survey, and 4,462 patients completed the survey (99.5%). 549 patients (12.2%) reported running preoperatively (53.4% THA, 39.7% TKA, 6.9% UKA). These preoperative runners had a mean ± standard deviation age of 61.3 ± 11.4 years and a mean BMI of 26.9 ± 4.6 kg/m^2^, and 36.3% were female (Table 1). Follow-up after TJA in the cohort of patients who ran preoperatively and postoperatively ranged from 1 year (41.2%), 2 years (17.2%), 3 to 5 years (9.7%), 5 to 10 years (3.0%), to >10 years (3.4%).

**Table 1:** Demographics of THA, TKA, and UKA Patients Who Completed the ReJoin Survey

	Runner Before Surgery	Runner Before and After Surgery	Not Runner Before Surgery	Not Runner Before TJA, but Runner After TJA	Total
Mean age^a^	61.27 (11.4)	58.60 (10.8)	65.78 (10.6)	61.3 (13.2)	65.22 (10.8)
Mean BMI^a^	26.93 (4.6)	25.61 (3.3)	29.73 (6.3)	27.1 (5.9)	29.38 (6.2)
Female	180 (36.3%)	17 (32.1%)	2161 (61.0%)	13 (61.0%)	2341 (55.7%)
Male	315 (63.5%)	36 (67.9%)	1383 (39.0%)	13 (50%)	1698 (40.3%)
Other	1 (0.2%)	0	1 (0.03%)	0	2 (0.05%)
Not reported	53 (9.7%)	12 (18.5%)	368 (9.4%)	14 (35%)	421 (8.7%)
UKA	38 (6.9%)	7 (10.8%)	151 (3.9%)	0	189 (4.2%)
THA	293 (53.4%)	49 (75.4%)	1692 (43.2%)	27 (67.5%)	1985 (44.5%)
TKA	218 (39.7%)	9 (13.8%)	2070 (52.9%)	13 (32.5%)	2228 (49.9%)
*Number of cases*	549 (12.2%)	65 (11.8%)	3913 (87.7%)	40 (1.0%)	4462

THA = total hip arthroplasty, TKA = total knee arthroplasty, UKA = unicompartmental knee arthroplasty

a Mean (SD), All other values are reported as absolute numbers (%)

Furthermore, 40 patients (1.0%) who did not run before surgery reported running after TJA (67.5% THA, 32.5% TKA). Follow-up after TJA ranged from 1 year (56.8%), two years (27.0%), to 3 to 5 years (16.2%).

Demographic differences were observed between patients who ran before surgery, those who ran before and after surgery, those who were not runners before surgery, and those who started running after surgery. Age and BMI were highest in patients who were not runners before surgery (*P* < 0.0001), but sex was not different between groups (*P* > 0.05).

### Preoperative Running and Cross-Training

The 549 preoperative runners had varying levels of preoperative running experience: 35% were occasional runners, 39% were recreational but regular runners, 17.1% were serious runners, and 8.5% were competitive runners. Of these runners, 1.7% reported 0 to 1 years of running before surgery, 10.2% had 2 to 5 years, 14.1% had 6 to 10 years, 15.8% had 11 to 15 years, 11.7% had 16 to 20 years, and 46.5% had >20 years. These patients also participated in weekly muscle strengthening activities, with 27.1% exercising 0 to 2 days per week, 52.5% exercising 3 to 5 days per week, and 15.3% exercising 6 to 7 days per week. Of the 40 patients who did not run before surgery but ran after TJA, 34.3% did muscle strengthening exercises 0 to 2 days per week, 54.3% exercised 3 to 5 days per week, and 11.4% exercised 6 to 7 days per week.

### Postoperative Running Practices

Most of the preoperative runners (70.0%) did not expect to return to running after surgery, whereas 30.5% did. Most patients (86.2%) who returned to running within 5 years after surgery did so within 1 year after surgery. Similarly, within the cohort of patients who did not run before surgery but ran after surgery, the majority (84.2%) began running within 1 year. THA patients (18%) were more likely to return to running compared with TKA patients (5%) (*P* < 0.0001).

Of all preoperative runners who returned to running, 20 (30.8%) reported pain with running after TJA and 45 (69.2%) did not, with detailed running frequencies in Tables 2 and 3. 67.2% of the preoperative runners who returned to running were satisfied with the amount of postoperative running, whereas 32.8% were not.

**Table 2:** Weekly Postoperative Running Frequency of THA, TKA, and UKA Patients Who Returned to Running

	Runner Before and After Surgery	Not Runner Before TJA, but Runner After TJA
0-2 days	45 (69.2%)	33 (89.2%)
3-5 days	17 (26.2%)	4 (10.8%)
6-7 days	3 (4.6%)	0
Not reported	0	3 (7.5%)
Total	65	40

THA = total hip arthroplasty, TKA = total knee arthroplasty, UKA = unicompartmental knee arthroplasty

**Table 3:** Weekly Postoperative Running Mileage of THA, TKA, and UKA Patients Who Returned to Running

	Runner Before and After Surgery	Not Runner Before TJA, but Runner After TJA
0-10 miles	49	35
11-15 miles	6	1
16-20 miles	3	0
21-25 miles	1	0
26-30 miles	1	0
31-35 miles	2	0
36-40 miles	2	0
Not reported	1	4
Total	65	40

THA = total hip arthroplasty, TKA = total knee arthroplasty, UKA = unicompartmental knee arthroplasty

Of the patients who did not run before surgery but ran after surgery, 8 (22.2%) reported pain with running after TJA and 28 (77.8%) did not, with lower running frequency (Table 2) and less mileage (Table 3) than preoperative runners. Patient satisfaction regarding postoperative running mileage was 85.7%, whereas 14.3% were not satisfied.

### Postoperative Revision Surgeries and Running Injuries

**Figure 1:**
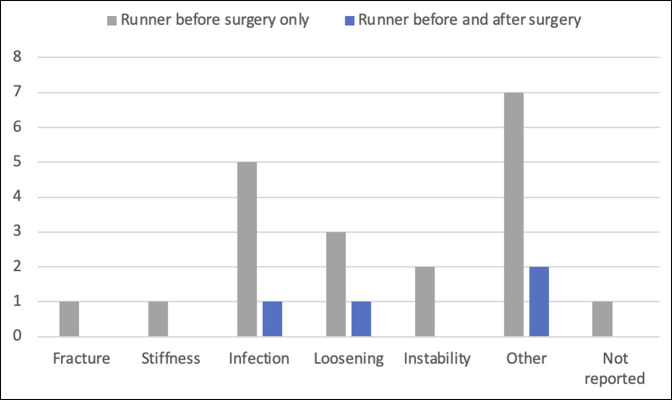
Graph showing reasons for revision surgeries for patients who ran before total hip arthroplasty, total knee arthroplasty, and unicompartmental knee arthroplasty.

Of the 549 preoperative runners, 481 reported on revision surgery status with 24 (5.0%) runners undergoing a revision surgery and 457 (95.0%) did not, and 20 of 416 (4.8%) preoperative runners who did not return to running postoperatively underwent a revision surgery (*P* = 0.55). Of the 65 runners who returned to running postoperatively, four underwent revision surgery (6.2%). Different reasons for revision surgery are shown in Figure 1.

Five patients (1 UKA, 4 THA) who returned to running after surgery reported a postoperative athletic injury, whereas 60 did not. The injuries were reported in the ankle (n = 1), knee (n = 2), calf (n = 1), and other (n = 1).

### Commitment to Exercise and Brief Resilience Scale

Preoperative runners who did not return to running (n = 416) had a mean BRS score of 3.8, which was similar to patients who started running postoperatively (n = 13) (Table 4). The 65 preoperative runners who returned to running postoperatively had a mean BRS score of 3.9.

**Table 4:** Brief Resilience Scores of THA, TKA, and UKA Patients

	Runner Before and After Surgery	Sample Size	Runner Before Surgery, but Did Not Return to Running	Sample Size	Not Runner Before TJA, but Runner After TJA	Sample Size
UKA	3.80 (0.5)	7	3.75 (0.6)	27	N/A	N/A
THA	4.03 (0.6)	49	3.85 (0.7)	220	3.77 (0.8)	27
TKA	3.26 (0.8)	9	3.77 (0.8)	169	3.96 (0.6)	13
Total	3.90 (0.7)	65	3.81 (0.8)	416	3.83 (0.8)	40

N/A = not applicable, THA = total hip arthroplasty, TKA = total knee arthroplasty, UKA = unicompartmental knee arthroplasty

Of the preoperative runners who did not return to running, 410 patients reported a mean CTE score of 40.5 ± 15.9 (Table 5). The 65 preoperative runners who returned to running postoperatively had a mean CTE score of 46.5 ± 17.6. Patients who were not runners preoperatively but started running after surgery (n = 13) had the lowest CTE score of 39.7 ± 19.8 (F[2,510] = 3.88, *P* = 0.02). No relationship was observed between BRS or CTE scores and postoperative running status for each procedure (*P* > 0.05).

**Table 5:** Commitment to Exercise Scores of THA, TKA, and UKA Patients

Runner Before and After Surgery	Sample Size	Runner Before Surgery, but Did Not Return to Running	Sample Size	Not Runner Before TJA, but Runner After TJA	Sample Size
53.43 (10.7)	7	45.04 (19.1)	25	N/A	N/A
45.00 (18.8)	49	40.43 (15.7)	217	40.65 (20.2)	27
49.00 (14.1)	9	39.92 (15.6)	168	37.50 (19.7)	13
46.46 (17.6)	65	40.50 (15.9)	410	39.66 (19.8)	40

N/A = not applicable, THA = total hip arthroplasty, TKA = total knee arthroplasty, UKA = unicompartmental knee arthroplasty

### Surgeon Recommendations

**Figure 2:**
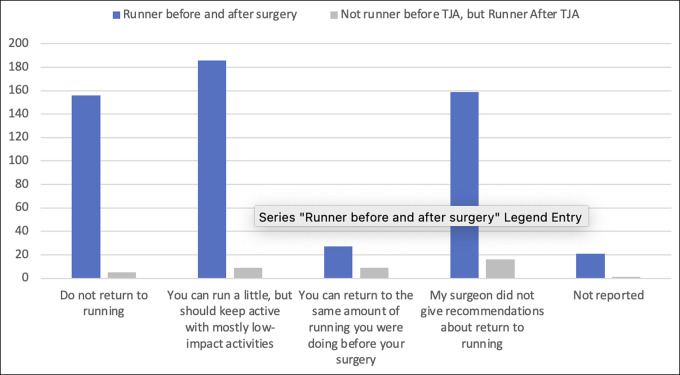
Graph showing surgeon recommendations for patients who return to running after total hip arthroplasty, total knee arthroplasty, and unicompartmental knee arthroplasty.

Among preoperative runners who returned to running and those who only ran after surgery, 28.4% of patients were told not to return to running after TJA, 34.4% were told they could run a little but should keep active mostly with low-impact activities, 6.3% were told they could return to the same amount of running they did before surgery, and 30.9% did not receive any recommendations about returning to running after TJA (Figure 2).

## Discussion

With more people undergoing TKA/THA/UKA to achieve the desired activity levels, standardized evidence-based recommendations for patients are needed to set realistic postoperative expectations. This study assessed a total of 4,462 TJA patients, of whom 12.2% self-reported running before surgery with 11.8% of these patients returning to running after TJA. For those who returned to running, 86.2% returned within 12 months after TJA. Notably, 40 patients (1.0%) who were not runners before TJA started running after TJA. Patient-reported surgeon recommendations were inconsistent because 30% did not receive a recommendation, and most patients were told they could run a little but should focus on low-impact activities. Preoperative runners who returned to running after surgery had the highest CTE scores.

Previous studies assessing high-impact activities after TJA have focused less specifically on running. Wylde et al^7^ found that 34.8% of patients were active in sports preoperatively, and 61.4% of these patients resumed sport participation postoperatively after primary TJA, UKA, or hip or patellar resurfacing. Our study reported a smaller percentage of active patients running, which is on par with other referenced studies.^7,9^ The literature is inconsistent regarding the frequency of high-impact activities after surgery; however, it is known that overall activity levels increase postoperatively.^14,15,16,17,18,19,20,21,22^ Therefore, it may be feasible to return to activities after TJA,^10^ as supported by this study.

Timing is also inconsistent for when surgeons recommend patients to start resuming activity postoperatively. In this study, >80% of patients reported running within one year after TJA. This is supported by timelines of returning to athletic sports 3 to 6 months after THA^15^ and 12 weeks for lower-impact activities after UKA.^23^ This study found a higher percentage of male patients who returned to running, which is comparable with other literature.^16^ Preoperative activity, BMI, and male sex also correlated with increased postoperative activity.^24^ One study assessing 160 TKA patients found that 49% of patients reported preoperative weekly athletic activity^9^ while another study reported 85% of TKA patients engaged in >1 recreational activity preoperatively.^14^ It has also been reported that THA patients are more likely to be able to return to activity postoperatively^16^ compared with TKA patients, consistent with the results of this study. Huch et al^16^ attributed a higher rate of return to postoperative activity in THA patients to more pain relief than TKA patients, which was also supported by Vogel et al.^20^ In our study, most of the preoperative runners who returned to running after TJA (69.2%) did not report pain while running, whereas 22.2% of the postoperative runners who did not run before surgery reported pain while running. Similarly, we found over three times more TKA patients reporting pain with postoperative running compared with THA patients. These results may support why a higher percentage of THA patients were able to return to running.

Resilience and commitment to exercise had not been assessed in TJA patients, and we found that preoperative runners who returned to running after TJA had the highest BRS and CTE scores compared with other patients, indicating stronger resilience and dependency on exercise. In other fields, the validated CTE markedly correlated with age, body fat percentage, obsessive-compulsiveness, and addictiveness.^12^ The BRS has been used in chronic pain studies to evaluate patients' ability to recover from stress.^13^ In our study, neither the BRS nor CTE scales were strong predictors of postoperative running activity. Future research on a larger sample size exploring this relationship may be beneficial.

Overall, expectations to run postoperatively were low. However, 67.2% of preoperative runners who returned to running were satisfied, and 85.7% of patients who did not run preoperatively were satisfied with the amount of postoperative running. Alternative studies shared similar results regarding pain relief and physical function being exceeded at 5 years after TKA.^25^ Research reported that patients returned to their desired high-impact activities after TKA and rated satisfaction as 9.1 of 10.^8^ Therefore, preoperative patient counseling regarding returning to running or other high-impact activities can benefit expectation management.

For postoperative injury and revision rates, preoperative runners who returned to running after TJA had greater self-reported injury and higher revision rates compared with preoperative runners who did not return to running, with the revision rates of 6.2% versus 4.8%, respectively. Although the difference in revision rates was not statistically significant, it is notable that larger sample size and longer follow-up is necessary to discern potential increasing revision trends with subsequent implant wear. There is concern that increased high-impact activity can lead to increased implant wear and higher revision rates.^10^ One study reported higher revision rates 10 years after THA with a history of high-impact sports than the low-impact activity cohort.^26^ Similarly, revision rates were higher in patients who were more active after THA (28%) compared with those who were not (6%).^27^ Conversely, another study found that revision rates in active patients after THA (1.6%) were lower than those without postoperative sport activity (14.3%).^28^ Similarly, patients who played sports after THA had lower revision rates because of prosthetic loosening (5%) compared with 10% in patients without sport activity.^29^ It is notable that several of the reported studies assessed patients who received first generation polyethylene liners^20^ while highly cross-linked polyethylene may withstand increased wear and kinematics of high-impact activity.^30-32^ Future research is needed to discern whether the use of materials such as cross-linked polyethylene in active TJA patients may affect revision rates.

As previously stated, there are no standardized surgeon recommendations for returning to running, or high-impact activities, to share with future TJA patients.^16,20,33^ Our study supported this finding because 30% of orthopaedic surgeons did not provide recommendations. For preoperative runners who returned to running after TJA, only 5% reported being told by their orthopaedic surgeon that they could return to the same level of preoperative running. However, because our study was based on patient recall of surgeon recommendations preoperatively, the accuracy of patient recall cannot be confirmed. A survey of the Hip Society members in 2005 revealed mixed recommendations on returning to high-impact and low-impact activities after THA, with specific recommendations to not run after TJA.^33^ Since 1995, a similar survey of the Knee Society showed that nine activities were permitted that were previously discouraged, showing how the recommendations have evolved.^33^ The Mayo Clinic survey revealed that of 28 common sports, only sailing, swimming laps, cycling, golfing, scuba diving, and bowling were recommended after TJA, and cross-country skiing was permitted after TKA. There was a consensus to forbid running, waterskiing, football, baseball, basketball, hockey, handball, karate, soccer, and racquetball after THA.^34^ Overall, restrictions on activities after THA were more lenient than those suggested after TKA.^20^

Although these findings contribute to the literature, our study is not without limitations. Because data were collected across multiple institutions, surgeon variability and potential geographic variations should be explored. Owing to the voluntary nature of this study, the patients did not answer all survey questions, accounting for differences in sample size for data measures. The relatively low incidence of runners limited our statistical power and analyses that could be completed. Furthermore, the survey data reflects self-reported runner status because the survey did not define the criteria of being a runner (e.g., current runner or mileage criteria). Additional data collection examining long-term outcomes is warranted to provide evidence-based recommendations on the safety of running after TJA.

In conclusion, 12% of TKA/THA/UKA patients ran preoperatively, and 11.8% were able to return to running postoperatively. Most patients returned to running <1 year after surgery, and markedly more THA patients ran postoperatively than TKA patients. Patients who returned to running after surgery had higher CTE and BRS scores, showing higher resilience and exercise dependence. Patient satisfaction was high for postoperative running ability, duration, and frequency. Interestingly, 1.0% patients who were not preoperative runners started running postoperatively. Preoperative runners who ran after surgery were more likely to undergo revision surgery compared with only postoperative runners. Patients who return to high-impact activities postoperatively should follow frequent follow-up protocols to assess implant wear and potential complications. Furthermore, surgeon recommendations to return to running after TJA were inconsistent, promoting the need for additional research and patient education for realistic recovery expectations.
